# Bad Plans

**Published:** 1859-09

**Authors:** 


### BAD PLANS.

It is a bad plan to sleep in the day-time, it prevents refresh ing sleep at night.

It is a bad plan to eat, "to make it even."

It is a bad plan to spit, or blow your nose on the side walk, it nauseates the passer-by, and may cause a broken limb.

It is a bad plan to run away from trouble, "face the music" bravely, and it will be largely shorn of its tall proportions.

It is a bad plan to eat when you are not hungry, for thereby you waste food, and bring suffering to yourself.

It is a bad plan to be always taking medicine, such persons are never well.

It is a bad plan for unprofessional people to read medical books, it first befogs and then befools.

It is a bad plan to begin the day in a fret, for it will be a day of happiness lost.

It is a bad plan to consult divers doctors at one time.

It is a bad plan to ride, when you could just as well walk.

